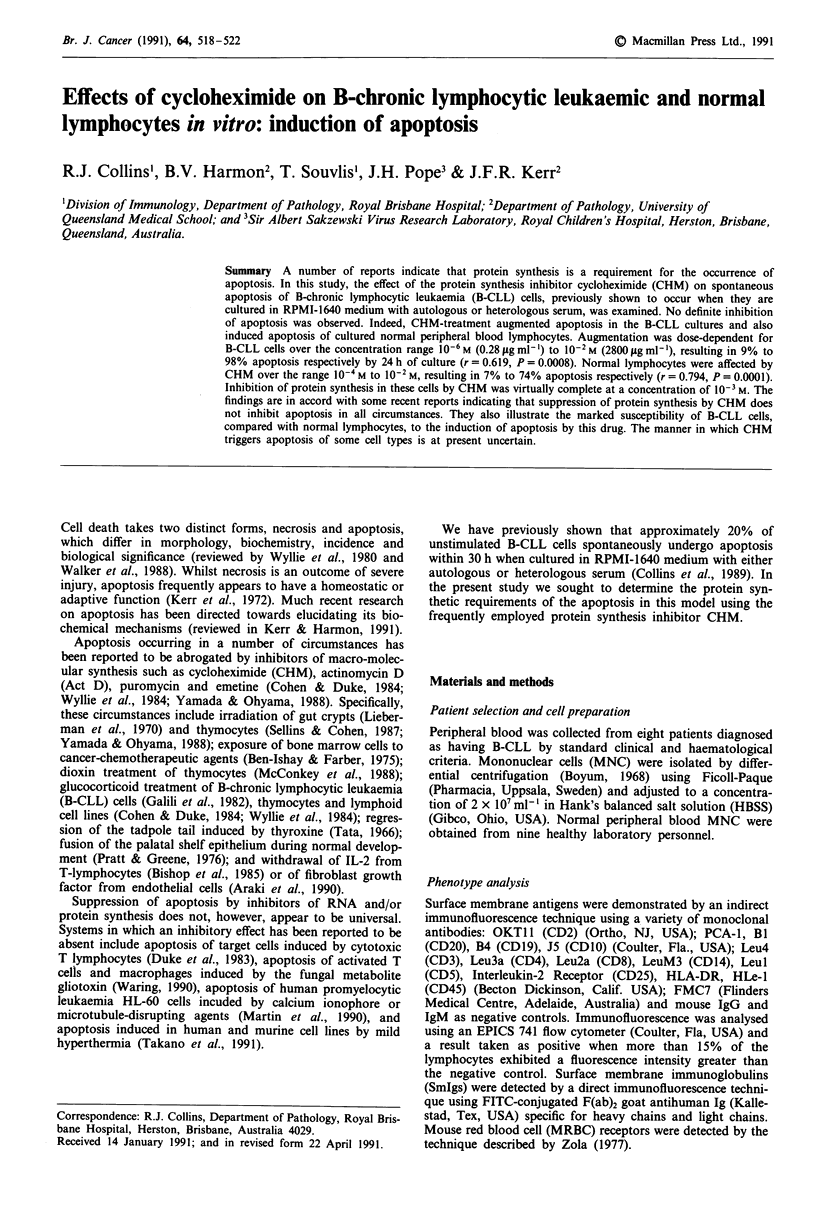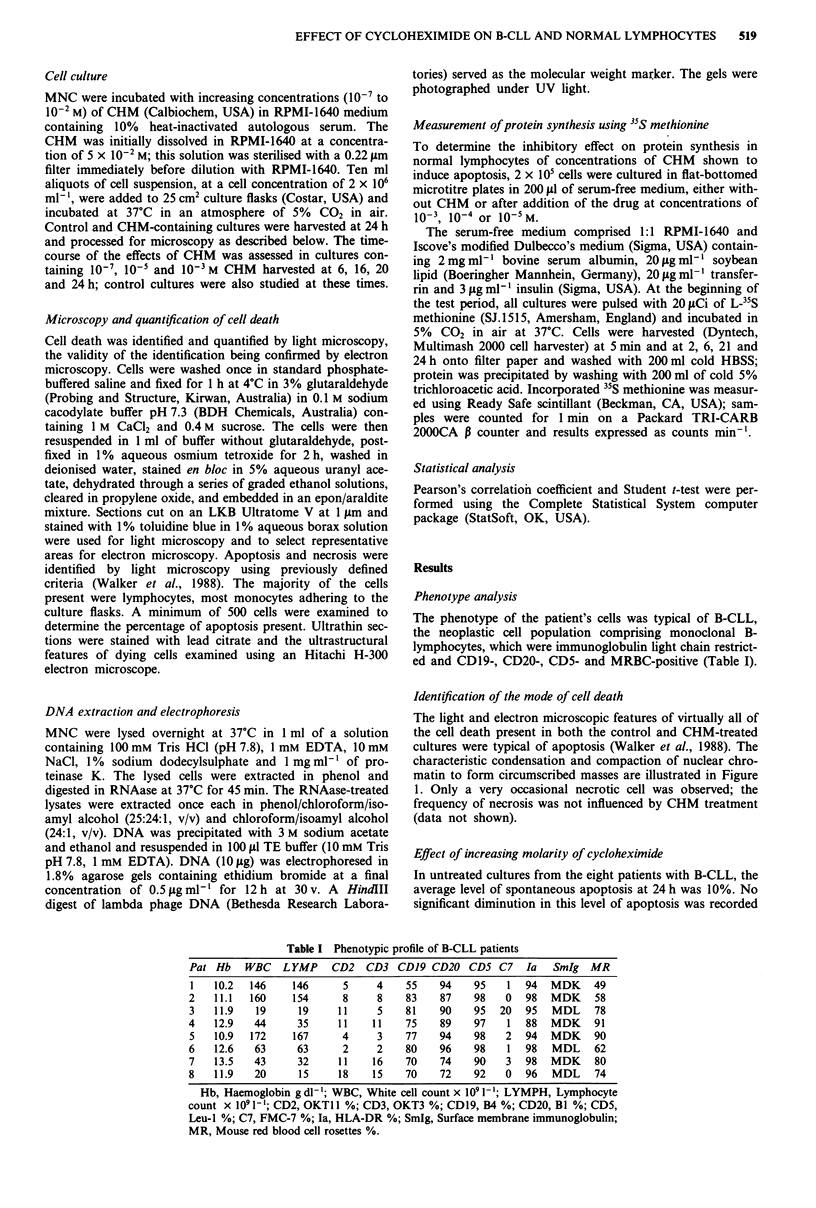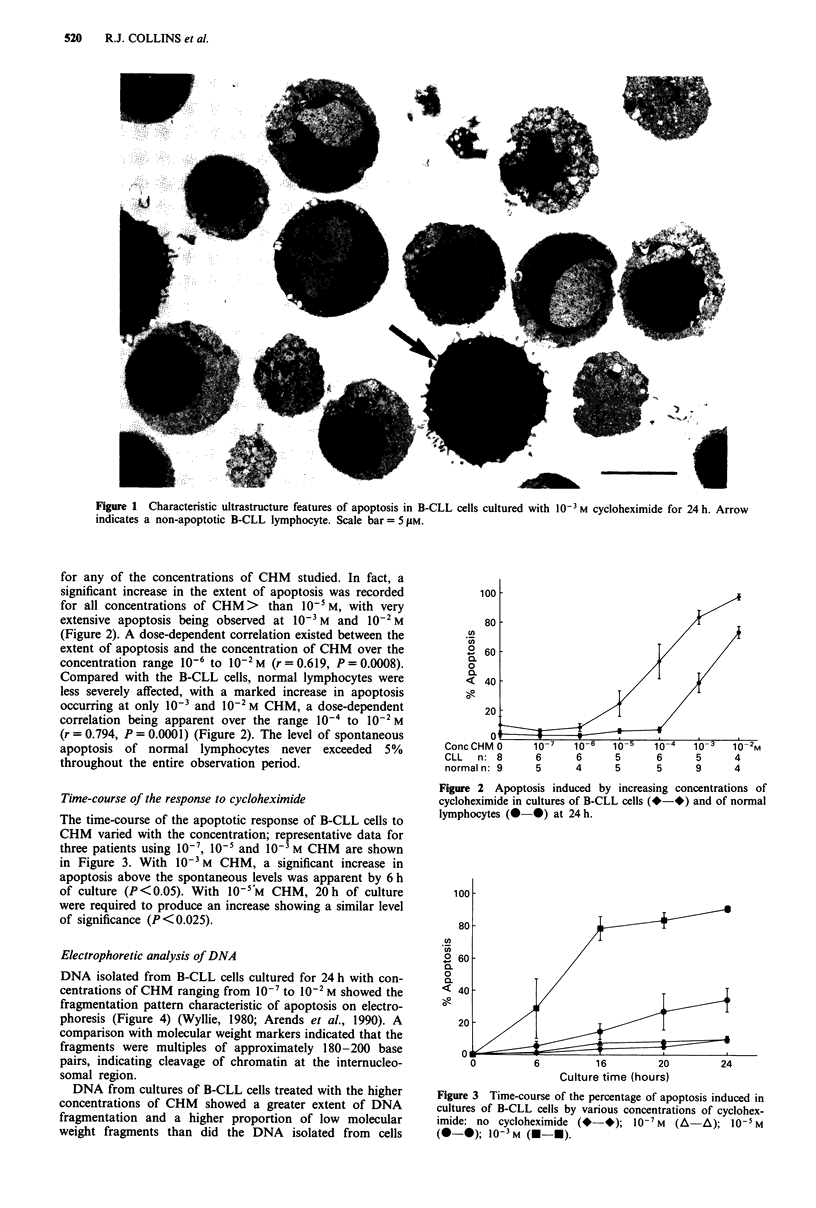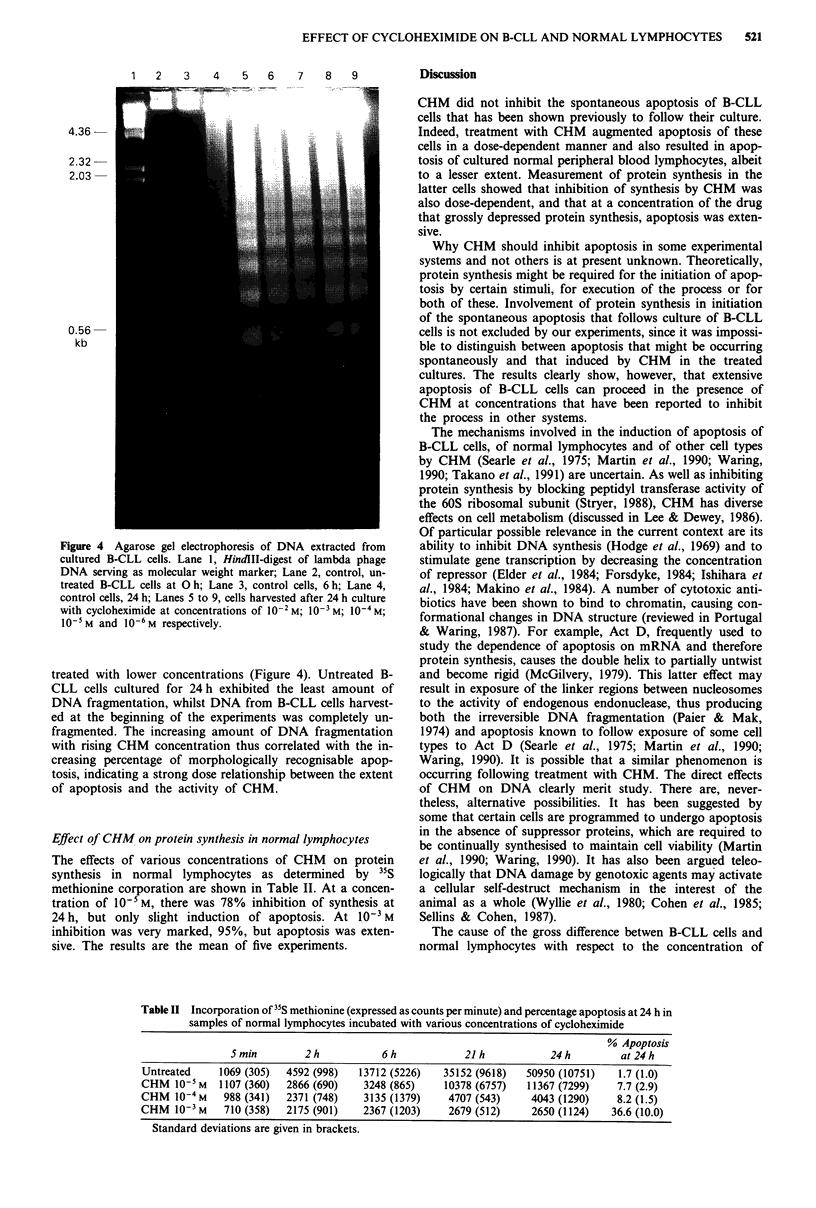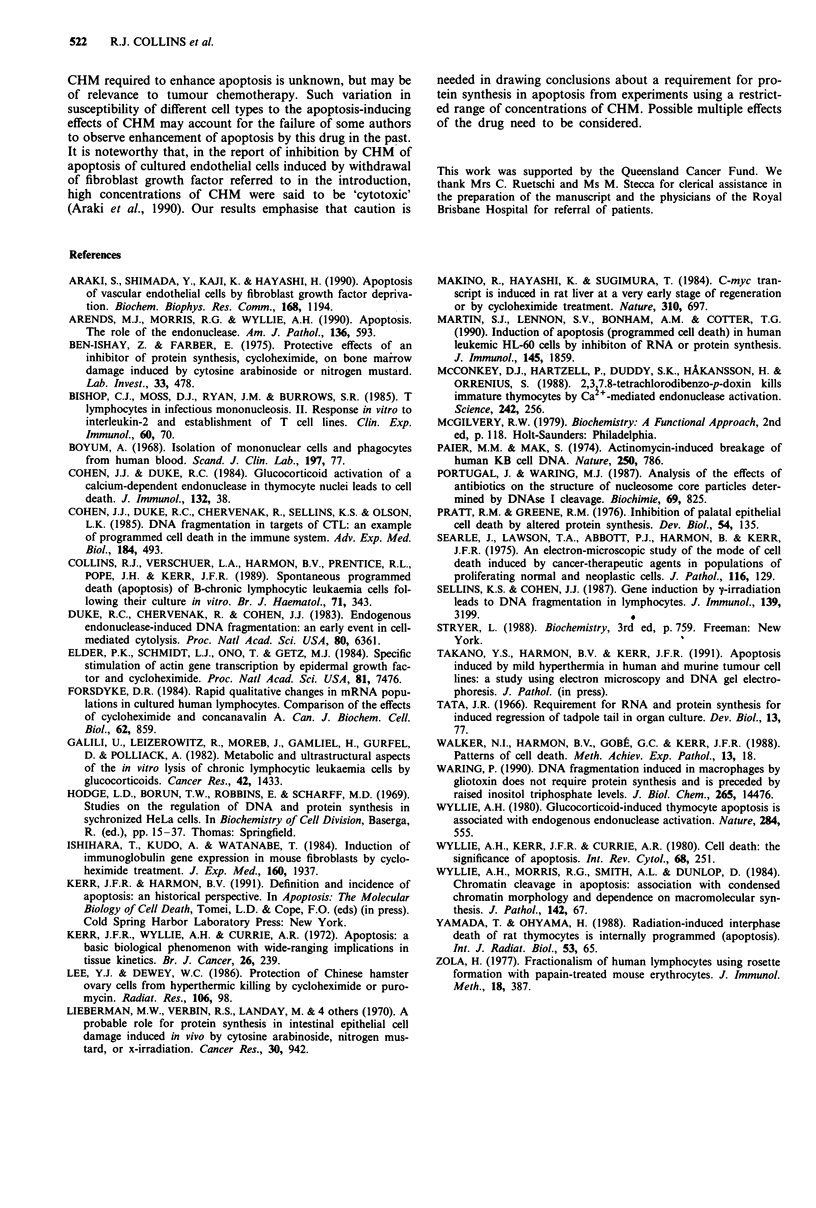# Effects of cycloheximide on B-chronic lymphocytic leukaemic and normal lymphocytes in vitro: induction of apoptosis.

**DOI:** 10.1038/bjc.1991.341

**Published:** 1991-09

**Authors:** R. J. Collins, B. V. Harmon, T. Souvlis, J. H. Pope, J. F. Kerr

**Affiliations:** Department of Pathology, Royal Brisbane Hospital, Herston, Queensland, Australia.

## Abstract

**Images:**


*Br. J. Cancer* (1991), **64**, 518–522 © Macmillan Press Ltd., 1991

### Effects of cycloheximide on B-chronic lymphocytic leukaemic and normal lymphocytes *in vitro*: induction of apoptosis

R.J. Collins[1], B.V. Harmon[2], T. Souvlis[1], J.H. Pope[3] & J.F.R. Kerr[2]

[1]*Division of Immunology, Department of Pathology, Royal Brisbane Hospital;* [2]*Department of Pathology, University of Queensland Medical School; and* [3]*Sir Albert Sakzewski Virus Research Laboratory, Royal Children's Hospital, Herston, Brisbane, Queensland, Australia.*

**Summary** A number of reports indicate that protein synthesis is a requirement for the occurrence of apoptosis. In this study, the effect of the protein synthesis inhibitor cycloheximide (CHM) on spontaneous apoptosis of B-chronic lymphocytic leukaemia (B-CLL) cells, previously shown to occur when they are cultured in RPMI-1640 medium with autologous or heterologous serum, was examined. No definite inhibition of apoptosis was observed. Indeed, CHM-treatment augmented apoptosis in the B-CLL cultures and also induced apoptosis of cultured normal peripheral blood lymphocytes. Augmentation was dose-dependent for B-CLL cells over the concentration range $10^{-6}$ M (0.28 μg $ml^{-1}$) to $10^{-2}$ M (2800 μg $ml^{-1}$), resulting in 9% to 98% apoptosis respectively by 24 h of culture ($r = 0.619$, $P = 0.0008$). Normal lymphocytes were affected by CHM over the range $10^{-4}$ M to $10^{-2}$ M, resulting in 7% to 74% apoptosis respectively ($r = 0.794$, $P = 0.0001$). Inhibition of protein synthesis in these cells by CHM was virtually complete at a concentration of $10^{-3}$ M. The findings are in accord with some recent reports indicating that suppression of protein synthesis by CHM does not inhibit apoptosis in all circumstances. They also illustrate the marked susceptibility of B-CLL cells, compared with normal lymphocytes, to the induction of apoptosis by this drug. The manner in which CHM triggers apoptosis of some cell types is at present uncertain.

Cell death takes two distinct forms, necrosis and apoptosis, which differ in morphology, biochemistry, incidence and biological significance (reviewed by Wyllie *et al.*, 1980 and Walker *et al.*, 1988). Whilst necrosis is an outcome of severe injury, apoptosis frequently appears to have a homeostatic or adaptive function (Kerr *et al.*, 1972). Much recent research on apoptosis has been directed towards elucidating its biochemical mechanisms (reviewed in Kerr & Harmon, 1991).

Apoptosis occurring in a number of circumstances has been reported to be abrogated by inhibitors of macro-molecular synthesis such as cycloheximide (CHM), actinomycin D (Act D), puromycin and emetine (Cohen & Duke, 1984; Wyllie *et al.*, 1984; Yamada & Ohyama, 1988). Specifically, these circumstances include irradiation of gut crypts (Lieberman *et al.*, 1970) and thymocytes (Sellins & Cohen, 1987; Yamada & Ohyama, 1988); exposure of bone marrow cells to cancer-chemotherapeutic agents (Ben-Ishay & Farber, 1975); dioxin treatment of thymocytes (McConkey *et al.*, 1988); glucocorticoid treatment of B-chronic lymphocytic leukaemia (B-CLL) cells (Galili *et al.*, 1982), thymocytes and lymphoid cell lines (Cohen & Duke, 1984; Wyllie *et al.*, 1984); regression of the tadpole tail induced by thyroxine (Tata, 1966); fusion of the palatal shelf epithelium during normal development (Pratt & Greene, 1976); and withdrawal of IL-2 from T-lymphocytes (Bishop *et al.*, 1985) or of fibroblast growth factor from endothelial cells (Araki *et al.*, 1990).

Suppression of apoptosis by inhibitors of RNA and/or protein synthesis does not, however, appear to be universal. Systems in which an inhibitory effect has been reported to be absent include apoptosis of target cells induced by cytotoxic T lymphocytes (Duke *et al.*, 1983), apoptosis of activated T cells and macrophages induced by the fungal metabolite gliotoxin (Waring, 1990), apoptosis of human promyelocytic leukaemia HL-60 cells incuded by calcium ionophore or microtubule-disrupting agents (Martin *et al.*, 1990), and apoptosis induced in human and murine cell lines by mild hyperthermia (Takano *et al.*, 1991).

Correspondence: R.J. Collins, Department of Pathology, Royal Brisbane Hospital, Herston, Brisbane, Australia 4029.
Received 14 January 1991; and in revised form 22 April 1991.

We have previously shown that approximately 20% of unstimulated B-CLL cells spontaneously undergo apoptosis within 30 h when cultured in RPMI-1640 medium with either autologous or heterologous serum (Collins *et al.*, 1989). In the present study we sought to determine the protein synthetic requirements of the apoptosis in this model using the frequently employed protein synthesis inhibitor CHM.

### Materials and methods

### *Patient selection and cell preparation*

Peripheral blood was collected from eight patients diagnosed as having B-CLL by standard clinical and haematological criteria. Mononuclear cells (MNC) were isolated by differential centrifugation (Boyum, 1968) using Ficoll-Paque (Pharmacia, Uppsala, Sweden) and adjusted to a concentration of $2 \times 10^7$ $ml^{-1}$ in Hank's balanced salt solution (HBSS) (Gibco, Ohio, USA). Normal peripheral blood MNC were obtained from nine healthy laboratory personnel.

### *Phenotype analysis*

Surface membrane antigens were demonstrated by an indirect immunofluorescence technique using a variety of monoclonal antibodies: OKT11 (CD2) (Ortho, NJ, USA); PCA-1, B1 (CD20), B4 (CD19), J5 (CD10) (Coulter, Fla., USA); Leu4 (CD3), Leu3a (CD4), Leu2a (CD8), LeuM3 (CD14), Leu1 (CD5), Interleukin-2 Receptor (CD25), HLA-DR, HLe-1 (CD45) (Becton Dickinson, Calif. USA); FMC7 (Flinders Medical Centre, Adelaide, Australia) and mouse IgG and IgM as negative controls. Immunofluorescence was analysed using an EPICS 741 flow cytometer (Coulter, Fla, USA) and a result taken as positive when more than 15% of the lymphocytes exhibited a fluorescence intensity greater than the negative control. Surface membrane immunoglobulins (SmIgs) were detected by a direct immunofluorescence technique using FITC-conjugated $F(ab)_2$ goat antihuman Ig (Kallestad, Tex, USA) specific for heavy chains and light chains. Mouse red blood cell (MRBC) receptors were detected by the technique described by Zola (1977).

*Cell culture*

MNC were incubated with increasing concentrations ($10^{-7}$ to $10^{-2}$ M) of CHM (Calbiochem, USA) in RPMI-1640 medium containing 10% heat-inactivated autologous serum. The CHM was initially dissolved in RPMI-1640 at a concentration of $5 \times 10^{-2}$ M; this solution was sterilised with a 0.22 μm filter immediately before dilution with RPMI-1640. Ten ml aliquots of cell suspension, at a cell concentration of $2 \times 10^6$ $ml^{-1}$, were added to 25 $cm^2$ culture flasks (Costar, USA) and incubated at 37°C in an atmosphere of 5% $CO_2$ in air. Control and CHM-containing cultures were harvested at 24 h and processed for microscopy as described below. The time-course of the effects of CHM was assessed in cultures containing $10^{-7}$, $10^{-5}$ and $10^{-3}$ M CHM harvested at 6, 16, 20 and 24 h; control cultures were also studied at these times.

*Microscopy and quantification of cell death*

Cell death was identified and quantified by light microscopy, the validity of the identification being confirmed by electron microscopy. Cells were washed once in standard phosphate-buffered saline and fixed for 1 h at 4°C in 3% glutaraldehyde (Probing and Structure, Kirwan, Australia) in 0.1 M sodium cacodylate buffer pH 7.3 (BDH Chemicals, Australia) containing 1 M $CaCl_2$ and 0.4 M sucrose. The cells were then resuspended in 1 ml of buffer without glutaraldehyde, post-fixed in 1% aqueous osmium tetroxide for 2 h, washed in deionised water, stained *en bloc* in 5% aqueous uranyl acetate, dehydrated through a series of graded ethanol solutions, cleared in propylene oxide, and embedded in an epon/araldite mixture. Sections cut on an LKB Ultratome V at 1 μm and stained with 1% toluidine blue in 1% aqueous borax solution were used for light microscopy and to select representative areas for electron microscopy. Apoptosis and necrosis were identified by light microscopy using previously defined criteria (Walker *et al.*, 1988). The majority of the cells present were lymphocytes, most monocytes adhering to the culture flasks. A minimum of 500 cells were examined to determine the percentage of apoptosis present. Ultrathin sections were stained with lead citrate and the ultrastructural features of dying cells examined using an Hitachi H-300 electron microscope.

*DNA extraction and electrophoresis*

MNC were lysed overnight at 37°C in 1 ml of a solution containing 100 mM Tris HCl (pH 7.8), 1 mM EDTA, 10 mM NaCl, 1% sodium dodecylsulphate and 1 mg $ml^{-1}$ of proteinase K. The lysed cells were extracted in phenol and digested in RNAase at 37°C for 45 min. The RNAase-treated lysates were extracted once each in phenol/chloroform/isoamyl alcohol (25:24:1, v/v) and chloroform/isoamyl alcohol (24:1, v/v). DNA was precipitated with 3 M sodium acetate and ethanol and resuspended in 100 μl TE buffer (10 mM Tris pH 7.8, 1 mM EDTA). DNA (10 μg) was electrophoresed in 1.8% agarose gels containing ethidium bromide at a final concentration of 0.5 μg $ml^{-1}$ for 12 h at 30 v. A *Hind*III digest of lambda phage DNA (Bethesda Research Laboratories) served as the molecular weight marker. The gels were photographed under UV light.

*Measurement of protein synthesis using $^{35}S$ methionine*

To determine the inhibitory effect on protein synthesis in normal lymphocytes of concentrations of CHM shown to induce apoptosis, $2 \times 10^5$ cells were cultured in flat-bottomed microtitre plates in 200 μl of serum-free medium, either without CHM or after addition of the drug at concentrations of $10^{-3}$, $10^{-4}$ or $10^{-5}$ M.

The serum-free medium comprised 1:1 RPMI-1640 and Iscove's modified Dulbecco's medium (Sigma, USA) containing 2 mg $ml^{-1}$ bovine serum albumin, 20 μg $ml^{-1}$ soybean lipid (Boeringher Mannhein, Germany), 20 μg $ml^{-1}$ transferrin and 3 μg $ml^{-1}$ insulin (Sigma, USA). At the beginning of the test period, all cultures were pulsed with 20 μCi of L-$^{35}S$ methionine (SJ.1515, Amersham, England) and incubated in 5% $CO_2$ in air at 37°C. Cells were harvested (Dyntech, Multimash 2000 cell harvester) at 5 min and at 2, 6, 21 and 24 h onto filter paper and washed with 200 ml cold HBSS; protein was precipitated by washing with 200 ml of cold 5% trichloroacetic acid. Incorporated $^{35}S$ methionine was measured using Ready Safe scintillant (Beckman, CA, USA); samples were counted for 1 min on a Packard TRI-CARB 2000CA β counter and results expressed as counts $min^{-1}$.

*Statistical analysis*

Pearson's correlation coefficient and Student *t*-test were performed using the Complete Statistical System computer package (StatSoft, OK, USA).

### Results

*Phenotype analysis*

The phenotype of the patient's cells was typical of B-CLL, the neoplastic cell population comprising monoclonal B-lymphocytes, which were immunoglobulin light chain restricted and CD19-, CD20-, CD5- and MRBC-positive (Table I).

*Identification of the mode of cell death*

The light and electron microscopic features of virtually all of the cell death present in both the control and CHM-treated cultures were typical of apoptosis (Walker *et al.*, 1988). The characteristic condensation and compaction of nuclear chromatin to form circumscribed masses are illustrated in Figure 1. Only a very occasional necrotic cell was observed; the frequency of necrosis was not influenced by CHM treatment (data not shown).

*Effect of increasing molarity of cycloheximide*

In untreated cultures from the eight patients with B-CLL, the average level of spontaneous apoptosis at 24 h was 10%. No significant diminution in this level of apoptosis was recorded

**Table I** Phenotypic profile of B-CLL patients

| *Pat* | *Hb* | *WBC* | *LYMP* | *CD2* | *CD3* | *CD19* | *CD20* | *CD5* | *C7* | *Ia* | *SmIg* | *MR* |
|---|---|---|---|---|---|---|---|---|---|---|---|---|
| 1 | 10.2 | 146 | 146 | 5 | 4 | 55 | 94 | 95 | 1 | 94 | MDK | 49 |
| 2 | 11.1 | 160 | 154 | 8 | 8 | 83 | 87 | 98 | 0 | 98 | MDK | 58 |
| 3 | 11.9 | 19 | 19 | 11 | 5 | 81 | 90 | 95 | 20 | 95 | MDL | 78 |
| 4 | 12.9 | 44 | 35 | 11 | 11 | 75 | 89 | 97 | 1 | 88 | MDK | 91 |
| 5 | 10.9 | 172 | 167 | 4 | 3 | 77 | 94 | 98 | 2 | 94 | MDK | 90 |
| 6 | 12.6 | 63 | 63 | 2 | 2 | 80 | 96 | 98 | 1 | 98 | MDL | 62 |
| 7 | 13.5 | 43 | 32 | 11 | 16 | 70 | 74 | 90 | 3 | 98 | MDK | 80 |
| 8 | 11.9 | 20 | 15 | 18 | 15 | 70 | 72 | 92 | 0 | 96 | MDL | 74 |

Hb, Haemoglobin g $dl^{-1}$; WBC, White cell count $\times 10^9 l^{-1}$; LYMPH, Lymphocyte count $\times 10^9 l^{-1}$; CD2, OKT11 %; CD3, OKT3 %; CD19, B4 %; CD20, B1 %; CD5, Leu-1 %; C7, FMC-7 %; Ia, HLA-DR %; SmIg, Surface membrane immunoglobulin; MR, Mouse red blood cell rosettes %.

**Figure 1** Characteristic ultrastructure features of apoptosis in B-CLL cells cultured with $10^{-3}$ M cycloheximide for 24 h. Arrow indicates a non-apoptotic B-CLL lymphocyte. Scale bar = 5 μM.

for any of the concentrations of CHM studied. In fact, a significant increase in the extent of apoptosis was recorded for all concentrations of CHM > than $10^{-5}$ M, with very extensive apoptosis being observed at $10^{-3}$ M and $10^{-2}$ M (Figure 2). A dose-dependent correlation existed between the extent of apoptosis and the concentration of CHM over the concentration range $10^{-6}$ to $10^{-2}$ M ($r = 0.619$, $P = 0.0008$). Compared with the B-CLL cells, normal lymphocytes were less severely affected, with a marked increase in apoptosis occurring at only $10^{-3}$ and $10^{-2}$ M CHM, a dose-dependent correlation being apparent over the range $10^{-4}$ to $10^{-2}$ M ($r = 0.794$, $P = 0.0001$) (Figure 2). The level of spontaneous apoptosis of normal lymphocytes never exceeded 5% throughout the entire observation period.

*Time-course of the response to cycloheximide*

The time-course of the apoptotic response of B-CLL cells to CHM varied with the concentration; representative data for three patients using $10^{-7}$, $10^{-5}$ and $10^{-3}$ M CHM are shown in Figure 3. With $10^{-3}$ M CHM, a significant increase in apoptosis above the spontaneous levels was apparent by 6 h of culture ($P < 0.05$). With $10^{-5}$ M CHM, 20 h of culture were required to produce an increase showing a similar level of significance ($P < 0.025$).

*Electrophoretic analysis of DNA*

DNA isolated from B-CLL cells cultured for 24 h with concentrations of CHM ranging from $10^{-7}$ to $10^{-2}$ M showed the fragmentation pattern characteristic of apoptosis on electrophoresis (Figure 4) (Wyllie, 1980; Arends *et al.*, 1990). A comparison with molecular weight markers indicated that the fragments were multiples of approximately 180–200 base pairs, indicating cleavage of chromatin at the internucleosomal region.

DNA from cultures of B-CLL cells treated with the higher concentrations of CHM showed a greater extent of DNA fragmentation and a higher proportion of low molecular weight fragments than did the DNA isolated from cells

**Figure 2** Apoptosis induced by increasing concentrations of cycloheximide in cultures of B-CLL cells (◆—◆) and of normal lymphocytes (●—●) at 24 h.

**Figure 3** Time-course of the percentage of apoptosis induced in cultures of B-CLL cells by various concentrations of cycloheximide: no cycloheximide (◆—◆); $10^{-7}$ M (Δ—Δ); $10^{-5}$ M (●—●); $10^{-3}$ M (■—■).

**Figure 4** Agarose gel electrophoresis of DNA extracted from cultured B-CLL cells. Lane 1, *Hind*III-digest of lambda phage DNA serving as molecular weight marker; Lane 2, control, untreated B-CLL cells at O h; Lane 3, control cells, 6 h; Lane 4, control cells, 24 h; Lanes 5 to 9, cells harvested after 24 h culture with cycloheximide at concentrations of $10^{-2}$ M; $10^{-3}$ M; $10^{-4}$ M; $10^{-5}$ M and $10^{-6}$ M respectively.

treated with lower concentrations (Figure 4). Untreated B-CLL cells cultured for 24 h exhibited the least amount of DNA fragmentation, whilst DNA from B-CLL cells harvested at the beginning of the experiments was completely unfragmented. The increasing amount of DNA fragmentation with rising CHM concentration thus correlated with the increasing percentage of morphologically recognisable apoptosis, indicating a strong dose relationship between the extent of apoptosis and the activity of CHM.

### *Effect of CHM on protein synthesis in normal lymphocytes*

The effects of various concentrations of CHM on protein synthesis in normal lymphocytes as determined by $^{35}$S methionine corporation are shown in Table II. At a concentration of $10^{-5}$ M, there was 78% inhibition of synthesis at 24 h, but only slight induction of apoptosis. At $10^{-3}$ M inhibition was very marked, 95%, but apoptosis was extensive. The results are the mean of five experiments.

### Discussion

CHM did not inhibit the spontaneous apoptosis of B-CLL cells that has been shown previously to follow their culture. Indeed, treatment with CHM augmented apoptosis of these cells in a dose-dependent manner and also resulted in apoptosis of cultured normal peripheral blood lymphocytes, albeit to a lesser extent. Measurement of protein synthesis in the latter cells showed that inhibition of synthesis by CHM was also dose-dependent, and that at a concentration of the drug that grossly depressed protein synthesis, apoptosis was extensive.

Why CHM should inhibit apoptosis in some experimental systems and not others is at present unknown. Theoretically, protein synthesis might be required for the initiation of apoptosis by certain stimuli, for execution of the process or for both of these. Involvement of protein synthesis in initiation of the spontaneous apoptosis that follows culture of B-CLL cells is not excluded by our experiments, since it was impossible to distinguish between apoptosis that might be occurring spontaneously and that induced by CHM in the treated cultures. The results clearly show, however, that extensive apoptosis of B-CLL cells can proceed in the presence of CHM at concentrations that have been reported to inhibit the process in other systems.

The mechanisms involved in the induction of apoptosis of B-CLL cells, of normal lymphocytes and of other cell types by CHM (Searle *et al.*, 1975; Martin *et al.*, 1990; Waring, 1990; Takano *et al.*, 1991) are uncertain. As well as inhibiting protein synthesis by blocking peptidyl transferase activity of the 60S ribosomal subunit (Stryer, 1988), CHM has diverse effects on cell metabolism (discussed in Lee & Dewey, 1986). Of particular possible relevance in the current context are its ability to inhibit DNA synthesis (Hodge *et al.*, 1969) and to stimulate gene transcription by decreasing the concentration of repressor (Elder *et al.*, 1984; Forsdyke, 1984; Ishihara *et al.*, 1984; Makino *et al.*, 1984). A number of cytotoxic antibiotics have been shown to bind to chromatin, causing conformational changes in DNA structure (reviewed in Portugal & Waring, 1987). For example, Act D, frequently used to study the dependence of apoptosis on mRNA and therefore protein synthesis, causes the double helix to partially untwist and become rigid (McGilvery, 1979). This latter effect may result in exposure of the linker regions between nucleosomes to the activity of endogenous endonuclease, thus producing both the irreversible DNA fragmentation (Paier & Mak, 1974) and apoptosis known to follow exposure of some cell types to Act D (Searle *et al.*, 1975; Martin *et al.*, 1990; Waring, 1990). It is possible that a similar phenomenon is occurring following treatment with CHM. The direct effects of CHM on DNA clearly merit study. There are, nevertheless, alternative possibilities. It has been suggested by some that certain cells are programmed to undergo apoptosis in the absence of suppressor proteins, which are required to be continually synthesised to maintain cell viability (Martin *et al.*, 1990; Waring, 1990). It has also been argued teleologically that DNA damage by genotoxic agents may activate a cellular self-destruct mechanism in the interest of the animal as a whole (Wyllie *et al.*, 1980; Cohen *et al.*, 1985; Sellins & Cohen, 1987).

The cause of the gross difference betwen B-CLL cells and normal lymphocytes with respect to the concentration of

**Table II** Incorporation of $^{35}$S methionine (expressed as counts per minute) and percentage apoptosis at 24 h in samples of normal lymphocytes incubated with various concentrations of cycloheximide

| | *5 min* | *2 h* | *6 h* | *21 h* | *24 h* | *% Apoptosis at 24 h* |
|---|---|---|---|---|---|---|
| Untreated | 1069 (305) | 4592 (998) | 13712 (5226) | 35152 (9618) | 50950 (10751) | 1.7 (1.0) |
| CHM $10^{-5}$ M | 1107 (360) | 2866 (690) | 3248 (865) | 10378 (6757) | 11367 (7299) | 7.7 (2.9) |
| CHM $10^{-4}$ M | 988 (341) | 2371 (748) | 3135 (1379) | 4707 (543) | 4043 (1290) | 8.2 (1.5) |
| CHM $10^{-3}$ M | 710 (358) | 2175 (901) | 2367 (1203) | 2679 (512) | 2650 (1124) | 36.6 (10.0) |

Standard deviations are given in brackets.

CHM required to enhance apoptosis is unknown, but may be of relevance to tumour chemotherapy. Such variation in susceptibility of different cell types to the apoptosis-inducing effects of CHM may account for the failure of some authors to observe enhancement of apoptosis by this drug in the past. It is noteworthy that, in the report of inhibition by CHM of apoptosis of cultured endothelial cells induced by withdrawal of fibroblast growth factor referred to in the introduction, high concentrations of CHM were said to be 'cytotoxic' (Araki et al., 1990). Our results emphasise that caution is needed in drawing conclusions about a requirement for protein synthesis in apoptosis from experiments using a restricted range of concentrations of CHM. Possible multiple effects of the drug need to be considered.

This work was supported by the Queensland Cancer Fund. We thank Mrs C. Ruetschi and Ms M. Stecca for clerical assistance in the preparation of the manuscript and the physicians of the Royal Brisbane Hospital for referral of patients.

### References

ARAKI, S., SHIMADA, Y., KAJI, K. & HAYASHI, H. (1990). Apoptosis of vascular endothelial cells by fibroblast growth factor deprivation. *Biochem. Biophys. Res. Comm.*, **168**, 1194.

ARENDS, M.J., MORRIS, R.G. & WYLLIE, A.H. (1990). Apoptosis. The role of the endonuclease. *Am. J. Pathol.*, **136**, 593.

BEN-ISHAY, Z. & FARBER, E. (1975). Protective effects of an inhibitor of protein synthesis, cycloheximide, on bone marrow damage induced by cytosine arabinoside or nitrogen mustard. *Lab. Invest.*, **33**, 478.

BISHOP, C.J., MOSS, D.J., RYAN, J.M. & BURROWS, S.R. (1985). T lymphocytes in infectious mononucleosis. II. Response *in vitro* to interleukin-2 and establishment of T cell lines. *Clin. Exp. Immunol.*, **60**, 70.

BOYUM, A. (1968). Isolation of mononuclear cells and phagocytes from human blood. *Scand. J. Clin. Lab.*, **197**, 77.

COHEN, J.J. & DUKE, R.C. (1984). Glucocorticoid activation of a calcium-dependent endonuclease in thymocyte nuclei leads to cell death. *J. Immunol.*, **132**, 38.

COHEN, J.J., DUKE, R.C., CHERVENAK, R., SELLINS, K.S. & OLSON, L.K. (1985). DNA fragmentation in targets of CTL: an example of programmed cell death in the immune system. *Adv. Exp. Med. Biol.*, **184**, 493.

COLLINS, R.J., VERSCHUER, L.A., HARMON, B.V., PRENTICE, R.L., POPE, J.H. & KERR, J.F.R. (1989). Spontaneous programmed death (apoptosis) of B-chronic lymphocytic leukaemia cells following their culture *in vitro*. *Br. J. Haematol.*, **71**, 343.

DUKE, R.C., CHERVENAK, R. & COHEN, J.J. (1983). Endogenous endonuclease-induced DNA fragmentation: an early event in cell-mediated cytolysis. *Proc. Natl Acad. Sci. USA*, **80**, 6361.

ELDER, P.K., SCHMIDT, L.J., ONO, T. & GETZ, M.J. (1984). Specific stimulation of actin gene transcription by epidermal growth factor and cycloheximide. *Proc. Natl Acad. Sci. USA*, **81**, 7476.

FORSDYKE, D.R. (1984). Rapid qualitative changes in mRNA populations in cultured human lymphocytes. Comparison of the effects of cycloheximide and concanavalin A. *Can. J. Biochem. Cell. Biol.*, **62**, 859.

GALILI, U., LEIZEROWITZ, R., MOREB, J., GAMLIEL, H., GURFEL, D. & POLLIACK, A. (1982). Metabolic and ultrastructural aspects of the *in vitro* lysis of chronic lymphocytic leukaemia cells by glucocorticoids. *Cancer Res.*, **42**, 1433.

HODGE, L.D., BORUN, T.W., ROBBINS, E. & SCHARFF, M.D. (1969). Studies on the regulation of DNA and protein synthesis in sychronized HeLa cells. In *Biochemistry of Cell Division*, Baserga, R. (ed.), pp. 15–37. Thomas: Springfield.

ISHIHARA, T., KUDO, A. & WATANABE, T. (1984). Induction of immunoglobulin gene expression in mouse fibroblasts by cycloheximide treatment. *J. Exp. Med.*, **160**, 1937.

KERR, J.F.R. & HARMON, B.V. (1991). Definition and incidence of apoptosis: an historical perspective. In *Apoptosis: The Molecular Biology of Cell Death*, Tomei, L.D. & Cope, F.O. (eds) (in press). Cold Spring Harbor Laboratory Press: New York.

KERR, J.F.R., WYLLIE, A.H. & CURRIE, A.R. (1972). Apoptosis: a basic biological phenomenon with wide-ranging implications in tissue kinetics. *Br. J. Cancer*, **26**, 239.

LEE, Y.J. & DEWEY, W.C. (1986). Protection of Chinese hamster ovary cells from hyperthermic killing by cycloheximide or puromycin. *Radiat. Res.*, **106**, 98.

LIEBERMAN, M.W., VERBIN, R.S., LANDAY, M. & 4 others (1970). A probable role for protein synthesis in intestinal epithelial cell damage induced *in vivo* by cytosine arabinoside, nitrogen mustard, or x-irradiation. *Cancer Res.*, **30**, 942.

MAKINO, R., HAYASHI, K. & SUGIMURA, T. (1984). C-*myc* transcript is induced in rat liver at a very early stage of regeneration or by cycloheximide treatment. *Nature*, **310**, 697.

MARTIN, S.J., LENNON, S.V., BONHAM, A.M. & COTTER, T.G. (1990). Induction of apoptosis (programmed cell death) in human leukemic HL-60 cells by inhibiton of RNA or protein synthesis. *J. Immunol.*, **145**, 1859.

MCCONKEY, D.J., HARTZELL, P., DUDDY, S.K., HÅKANSSON, H. & ORRENIUS, S. (1988). 2,3,7.8-tetrachlorodibenzo-*p*-doxin kills immature thymocytes by $Ca^{2+}$-mediated endonuclease activation. *Science*, **242**, 256.

MCGILVERY, R.W. (1979). *Biochemistry: A Functional Approach*, 2nd ed, p. 118. Holt-Saunders: Philadelphia.

PAIER, M.M. & MAK, S. (1974). Actinomycin-induced breakage of human KB cell DNA. *Nature*, **250**, 786.

PORTUGAL, J. & WARING, M.J. (1987). Analysis of the effects of antibiotics on the structure of nucleosome core particles determined by DNAse I cleavage. *Biochimie*, **69**, 825.

PRATT, R.M. & GREENE, R.M. (1976). Inhibition of palatal epithelial cell death by altered protein synthesis. *Dev. Biol.*, **54**, 135.

SEARLE, J., LAWSON, T.A., ABBOTT, P.J., HARMON, B. & KERR, J.F.R. (1975). An electron-microscopic study of the mode of cell death induced by cancer-therapeutic agents in populations of proliferating normal and neoplastic cells. *J. Pathol.*, **116**, 129.

SELLINS, K.S. & COHEN, J.J. (1987). Gene induction by γ-irradiation leads to DNA fragmentation in lymphocytes. *J. Immunol.*, **139**, 3199.

STRYER, L. (1988). *Biochemistry*, 3rd ed, p. 759. Freeman: New York.

TAKANO, Y.S., HARMON, B.V. & KERR, J.F.R. (1991). Apoptosis induced by mild hyperthermia in human and murine tumour cell lines: a study using electron microscopy and DNA gel electrophoresis. *J. Pathol.* (in press).

TATA, J.R. (1966). Requirement for RNA and protein synthesis for induced regression of tadpole tail in organ culture. *Dev. Biol.*, **13**, 77.

WALKER, N.I., HARMON, B.V., GOBÉ, G.C. & KERR, J.F.R. (1988). Patterns of cell death. *Meth. Achiev. Exp. Pathol.*, **13**, 18.

WARING, P. (1990). DNA fragmentation induced in macrophages by gliotoxin does not require protein synthesis and is preceded by raised inositol triphosphate levels. *J. Biol. Chem.*, **265**, 14476.

WYLLIE, A.H. (1980). Glucocorticoid-induced thymocyte apoptosis is associated with endogenous endonuclease activation. *Nature*, **284**, 555.

WYLLIE, A.H., KERR, J.F.R. & CURRIE, A.R. (1980). Cell death: the significance of apoptosis. *Int. Rev. Cytol.*, **68**, 251.

WYLLIE, A.H., MORRIS, R.G., SMITH, A.L. & DUNLOP, D. (1984). Chromatin cleavage in apoptosis: association with condensed chromatin morphology and dependence on macromolecular synthesis. *J. Pathol.*, **142**, 67.

YAMADA, T. & OHYAMA, H. (1988). Radiation-induced interphase death of rat thymocytes is internally programmed (apoptosis). *Int. J. Radiat. Biol.*, **53**, 65.

ZOLA, H. (1977). Fractionalism of human lymphocytes using rosette formation with papain-treated mouse erythrocytes. *J. Immunol. Meth.*, **18**, 387.